# Loot

**Published:** 1872-01

**Authors:** 


### Loot.

***Gelatinæ Medicatæ in Lamellis.***—By PROF. ALMEN of Upsala, Sweden. Translated by G. Treskastis, M.D.

Prof. Almen proposes to incorporate, for a more convenient administration, medicines in gelatine, made of water, glue and glycerine, or, when the medicine is not soluble in gelatine, gum arabic, or some other emulsive body. After the medicine has been thoroughly and uniformly mixed with the gelatine, it is spread evenly over a glass plate or slate with raised edges and with a perfectly even surface, which is divided by means of a file into 300 equal squares. In order to prevent the shrinking and sticking of the gelatine, the slate is greased slightly, while warm. In this manner the single squares can be easily separated from one another when dry, each containing an exact dose of the medicine.

About 6 grammes (one and a half drachms) of glue are required for 300 squares, and from 15 to 125 drops of glycerine, according to the solubility of the medicine, in order to render the gelatine as strong and flexible as paper.

If medicines are employed which do not dissolve in the preparation, calomel, for instance, a very viscid emulsion must be made in order to prevent the heavy drug from falling to the bottom. Gum arabic is best suited for this purpose. Sufficient water must be used to fill the slate up to the margin.

For the purpose of preparing good gelatine, 6 grammes (1½ drachms) of good glue are placed in 220–230 cc. (7 to 7½ oz.) of water and the necessary quantity of glycerine added. While the glue is dissolving, the apparatus is made ready and placed in a horizontal position by means of a spirit level.

After the glue is dissolved, the medicine is added, and dissolved by careful stirring. As soon as the liquid becomes lukewarm, it is poured over the plate as evenly as possible, and after a few hours the whole is transferred to a warm room, so as to prevent the mass from solidifying too rapidly.

After 24 hours the mass may be taken off the slate and divided.

The following are some of the formulas:

*Gelatinæ Morphiæ Acetatis.*—For 300 squares, it requires 36 drops of glycerine and 75 grains of acetate of morphine, each square containing ¼ grain of the salt.

*Gelatinæ Tartaris Emetici.*—37½ grs. of tartar emetic, 45 drops glycerine; ¼ gr. or 2 squares for a dose.

*Gelatinæ Cupri Sulphatis,* contain ½ gr. in each square; same dose as acetate of lead.

*Gelatinæ Opii.*—The powdered opium is rubbed up in water and well distributed through the mixture; or, better still, is suspended in an emulsion of gum arabic.

*Gelatinæ Extracti Opii.*—75 grs. extract of opium, 36 drops glycerine; each square contains ¼ gr. of extract.

*Gelatinæ Extracti Belladonnæ.*—The same as extract of opium.

*Gelatinæ Ipecac Pulveris.*—The usual quantity of glue is dissolved in water, with 100–130 drops of glycerine; 300 grains of powdered ipecac are added, and the mixture is warmed, and stirred continually until it becomes thick and thoroughly mixed.

*Gelatinæ Hydrarg. Chlor. Mitis.*—75 grs. of calomel are rubbed with 25 grs. of gum arabic, and a thick emulsion formed by the addition of a small quantity of water; to this the warm solution and 55 drops of glycerine are gradually added.

*Gelatinæ Ferri Carbonatis.*—An emulsion is made of carbonate of iron with mucilage of gum arabic, and the warm solution of glue is added, with 55 drops of glycerine. The sulphate, sesquichloride and iodide are not suitable.

*Gelatinæ Atropiæ Sulphatis.*—A warm solution, consisting of 1 grm. (16 grs.) of gelatine, 8 drops glycerine, and 75 c. c (2½ oz.) distilled water, is filtered through paper in a warm funnel, 3 grs. sulphate atropia are added, etc. Each square contains 1–100 gr. of the salt.

*Gelatinæ Extracti Physostigmatis,* (*Calabar Bean*) are prepared in the same manner.—*Schmidt's Jahrb., Aug.* 1871.

These gelatine squares need only to be floated on a tablespoonful of water to be swallowed easily by the patient.—[Ed.]—*Physician and Pharmacist, Nov.* 1871.

***Shall Eczema be Cured?***

The hesitation in curing eczema, lest it should "strike in," is thus met by Mr. Milton, in the *Medical Press and Circular*:

For years I have, in every instance, done my best to check the discharge of eczema as quickly as possible. During that period above 5,000 cases have passed under my notice, and as I have never seen or heard of any injurious results, I can only conclude that treatment cannot produce such an effect as bringing on internal disorder by relieving eczema. *Properly employed, treatment is neither innocuous or beneficial.* I can scarcely help thinking that, in such a large number of instances, if injurious results had been at all common, I must have heard something of them. On the other hand, it is quite certain that a number of patients, cured of profuse discharge, often of years' long duration, are, at the present time, not only well, but all the better for being freed from such a disgusting nuisance. I laid before the Medico Chirurgical Society the particulars of a case, where the discharge from an eczema, covering the leg from the calf to the sole of the foot, was so profuse that the patient, an old man in shattered health, said, that often, after a day's work, he returned home with his shoe half full of water. This state of things had gone on for three years, yet the speedy removal of it, so far from bringing on any internal affection, was followed by a decided improvement in the patient's health. The old man was very well known in the part of the city where he resided, near London Bridge, and some years after, when I last heard of him, was certainly quite as well as he had been previous to having the eczema. At the same time another instance was quoted, where a case of long standing eczema of the hand was cured, and where four years after the patient was in excellent health. This man, too, could have been easily identified, being a signal man at the Shoreditch station of the Great Eastern Railway. I could easily add to the list.

No doubt, if a patient suffering under eczema be attacked by some malady assailing the surface of the skin and the internal organization at the same time, as one of the ex-anthemata for instance, the eczema *may* be removed or suspended (for I trust I have shown that this does not certainly happen) as would many complaints, such as gonorrhœa; but I presume it would scarcely be considered the proceeding of a rational being to leave a gonorrhœa to take its own course, lest the removal of it might cause the development of some internal malady.

In all the cases I have seen, where eczema was complicated by an internal disorder such as bronchitis, an exacerbation of this, so far from relieving the eczema, either had no effect or made it worse; while in no case did the increased discharge, when the eczema was worse, in any way mitigate the internal affection. Thus, a poor weaver, suffering from eczema of the leg, came under my care. The disease of the skin was cured, and the patient remained well till an attack of bronchitis, at the beginning of the ensuing winter, prostrated him. In a very short time the eczema returned as bad as before, but without in the least relieving the bronchitis. A few years ago an old man came under my care for eczema of the leg. He was cured, and after an interval of quite four years, he again applied with the same complaint in both legs. I questioned him closely, and learned that he had fallen into bad health, that then the eczema came on, and that the worse it grew the worse he became in other respects—a statement quite borne out by the results of treatment, for the eczema disappeared as he improved in health. A poor woman was recently in attendance at St. John's Hospital who had been four times the subject of a bad attack of bronchitis; each time she was laid up in this way an old eczema of the ancle relapsed and passed into a state of ulceration. There is a middle-aged woman attending now at the same institution for eczema; she has twice suffered from bronchitis, and twice eczema has followed the coming on of the chest affection.

I could have added many more cases, but I need no longer note them down, as I have found no evidence on the other side of the question, and to heap together facts, merely to swell the bulk of testimony without adding to its real value, seems to me sheer waste of time. My experience is, that *if two or three cases will not induce men to think upon a question, two or three hundred will not.* I shall, therefore, content myself with adducing the evidence of M. Rayer, who supports the view I have been endeavoring to combat, as to there being a connection between the healing of an internal complaint and the cure of eczema. M. Rayer then says,* that he treated a patient for gastro-enteritis, who had been previously suffering from eczema, and that *during all the time the gastro intestinal inflammation lasted, the eczema was worse.* Again he says,† of another patient, "the appetite fell off remarkably (a certain sign that the health was not so good as formerly), an occurrence which was followed by a notable exacerbation of the eczematous affection."

I think, then, we may conclude that the fear of curing eczema, of however long standing it may be, and however delicate the health of the patient, *is not warranted by either proof or analogy;* that no known agent possesses the power of repelling eczema; that we can cure it only by means which improve the health at the same time; and that it is as justifiable to arrest its discharge as that of diarrhœa or cholera. And I may here remark, that all

* "Treatise on Diseases of the Skin." Translated by Willis. Second Edition, 1835. Page 316.

† Ibid. Page 322.

that has been said of eczema may be said of ulcer; there is no danger in healing it up, no bad symptoms ever arose from doing so. Those reported to have occurred were the offspring of prejudice or faulty observation, and offer only a too painful comment on the mode in which surgery has often been studied and taught.

***Sesquichloride of Iron as a Prophylactic of Acute Rheumatism.***—By Dr. Anstie.

A considerable space of time has now elased since the announcement by Dr. Russell Reynolds, of his observations on the successful treatment of acute rheumatism by large and frequent doses of the tincture of sesquichloride of iron. I do not know to what extent this plan of treatment has become generalized; but there have been a good many reports in the medical journals of its employment in different hospitals; and the balance of evidence derivable from these seems distinctly favorable to the method. My own experience of it in fully declared acute rheumatism has not been large. I have treated six cases altogether with the sesquichloride, and in four of these I think the results distinctly bore out the main assertions of Dr. Reynolds, as to the prompt relief of the pains, the limitation of the extent of the mischief, and the shortening of the illness; in the other two, the medicine seemed to have no special effect. But it is not of the use of the sesquichloride in the fully developed acute rheumatism that I now wish to speak. My opportunities of seeing disease on a large scale being chiefly those afforded by the out-patient room, it is rather the first advancings and threatenings of acute rheumatism, than the declared disease, that I am in the habit of seeing. A considerable number of persons present themselves in my out-patient room, during the course of twelve months, suffering from the preliminaries of acute rheumatism; it is one of the small group of really serious diseases (amongst a much larger variety of trivial complaints), which occupy one's attention in out-patient practice, and was formerly a matter of great dissatisfaction to me, from the apparently almost total failure of remedies to produce any effect. Whereas threatenings of gout could be very commonly dealt with in such a manner as to prevent the attack, or render it trivial, the onset of acute rheumatism seemed never to be averted by drugs when once the prodromata had reached the stage which pretty frequently presented itself before me, viz., a more or less obscure aching of several joints, a yellow sallowness of face, with patches or streaks of dusky redness, blanket-like furring of tongue, an oily moisture of skin, a distinct though slight elevation both of pulse and temperature, and a certain anxiety of respiration. So far as the history of such patients could be traced, they were almost invariably found to have developed the full symptoms of the acute disease, and very often (after once seeing them in the out-patient room), one encountered them, a few days later, in a ward of the hospital.

Very different have been the results of treatment since I adopted the use of full doses of sesquichloride of iron, from the first moment of such cases presenting themselves. During the past twelve months I have done this fully. Whenever a patient has presented himself with articular pain and slight fever, that were plainly of the rheumatic and not of the gouty type, he has been at once placed on thirty or forty minim doses of the tincture of sesquichloride, from three to six of which, according to the severity of the symptoms, have been given in each twenty-four hours. I have several times called the attention of the students to the fact, that (unlike what used to happen) these cases now reappear in my out-patient room on my next hospital day; and in the great majority of instances declare themselves greatly relieved.—*Medical Archives.*

### *Alkaline Sulphites in Marsh Fevers.*

M. Polli contributes a paper on this subject to the *Journal de Medecine*, in which he remarks that the nature of marsh miasms as well as their action on the organism will be greatly illuminated by the scientific contributions of those who have treated this important question at the medical congress of Florence in September, 1869. Malaria, he thinks, must be included in the class of causes which produce zymotic affections; and he now desires to direct attention to an anti-zymotic remedy, to the use of which he has been first led by scientific induction, and the value of which his clinical experience has not failed to demonstrate.

He conceived, as far back as 1861, the hope of being able to apply it in these cases, from finding that sulphurous acid enjoyed not only reducing properties but also antiseptic and antifermentative qualities, even when combined with the alkalies and the alkaline earths, and that in the state of sulphite it could be administered to animals in much larger doses than when free, without producing any of the inconveniences inseparable either from its inhalation in the gaseous state or from the injection of its more or less concentrated aqueous solution. Numerous experiments practiced on dogs having established the amount of the sulphite of soda, of the hyposulphite of soda, and of the sulphite of magnesia that could on the one hand be tolerated, and on the other was active, he at once proceeded to exhibit it in various diseases produced by the introduction into the blood of various septic and contagious matters, such as putrefying blood, decomposed pus, the fluid of glanders, etc. He then proceeded to determine by experiments instituted on himself and a friend, what doses of sulphite could be administered to man, with the object of prescribing it in cases of purulent affection, and in marsh fevers. His experiments have extended over several years, and have always been controlled or compared with quinine. The results he has arrived at are:

1. That marsh fever can be cured by the sulphites alone. 2. That the action of the sulphites is less rapid on the attack of the fever than the sulphate of quinine; they do not stop so suddenly the periodical course of the fever, but they usually gradually diminish the violence of the symptoms till they disappear altogether. 3. That the sulphites, *en ravanche*, act much more certainly in preventing the return of the fever than quinine. Amongst 403 cases treated by the sulphites, relapses only occurred in 5.7 per cent., whilst in 183 cases treated with sulphate of quinine the relapses amounted to 44.5 per cent. 4. That many cases of miasmatic fever, long rebellious under treatment by quinine, were cured by the sulphites alone. 5. That the sulphites can be employed with success even as a prophylactic means, and that they may be thus used for long periods without danger, which is not the case with the preparations of quinine. 6. That the sulphites can be administered without danger, in spite of concomitant gastro-intestinal irritation, and during the attack; and finally, that many sequelæ of fever (excepting always anæmia) may be very advantageously treated with the sulphites. Since the sulphites have cured marsh fevers as well and perfectly as quinine, and have been found even still more serviceable than quinine in yellow fever, it has been suggested that the febrifuge action of quinia may be due to an anti-fermentative action analogous to that which the sulphites exert on putrefying substances, and this view has been maintained and confirmed by M. Pavesi and M. Binz. It is not surprising now that carbolic acid, creosote, etc., have been used in similar cases with good effects. Arsenic perhaps acts in the same manner. The *curative* treatment adopted by M. Polli is given in the following prescriptions: If sulphite of soda be used, the proportion is 20 grammes of the salt in 200 of water, sweetened with honey or some aromatic syrup. This quantity is given in the course of twenty-four hours, in divided doses. When the sulphite of magnesia is prescribed, he gives 12 grammes in the same quantity of water, taken in four or six doses. When the hyposulphite of soda, 15 grammes in 300 of water, taken in similar manner. It is essential to take the remedy one hour before or two hours after a meal, and not to drink, except after a long interval, any acid substance, such as lemonade, or to take acid fruits or vinegar.

The *prophylactic* treatment.—For this purpose he prescribes 6 grammes of sulphite of magnesia, or 10 grammes of sulphite of soda, or 8 grammes of hyposulphite of soda, in two doses, dissolved in water, morning and evening, and considers this sufficient to preserve an adult during the season favorable for endemic disease.

This dose can be taken without inconvenience for several months together. M. Polli does not employ either the sulphite of potash or the sulphite of lime, the first having a disagreeable flavor and a too debilitating action; the latter being but little soluble, and having also a disagreeable flavor. The hyposulphate of lime may, however, be administered, and is very useful in certain phases of tuberculous phthisis.—(*Journal de Medecine, du Chirurgie, et de Pharmacologie, Oct.*, 1871.)—*London Practitioner.*

***Clinical Lecture.***—Clinical Lecture on those Symptoms in Women which point to the Expediency of instituting Local Examination. By ROBERT BARNES, M.D., Obstetric Physician to St. Thomas's Hospital, etc. etc.

There is nothing special in the mode of studying the diseases of women. Just as the ophthalmic surgeon is led to examine the eye because the patient complains of loss or disturbance of its function, or because he feels pain in it, or has some other subjective symptom referred to that organ, so by disturbances of function or some other subjective sign are we led to the discovery of disease of the sexual organs. When the function of an organ is disturbed, the *prima facie* inference is that the organ itself which constitutes the mechanism by which that function is performed is out of gear. This is not indeed always absolutely true; because an impaired state of the blood, or disordered innervation, or derangement of a different organ, may entail the functional disorder which arrests our attention. The genital organs are no exception to this proposition. The functions of the ovaries, uterus, or vagina, may be seriously deranged by a state of anæmia or blood-poisoning, by disease of the nervous centres, by disease of the heart, lungs, or liver. These functions may be even more seriously affected by mechanical pressure in contiguous parts. Still the fact remains that we can hardly appreciate rightly or successfully treat these primary or correlated diseases if we do not take into careful consideration the state of the genital organs themselves. The general or distant affections require to be investigated and treated; but it is not safe to overlook the organs that may be secondarily involved.

It is needless to say that every woman who is ill and seeks advice does not suffer from disorder of the sexual system. She may labor under various constitutional disorders, and under disorders of parts of the body quite independent of the sexual system. On the other hand, general or local disorders may in their course react upon, and induce disorders in, the sexual system. And there are disorders of this special system, commencing in it, and in their turn reacting upon, and inducing disorder in, distant organs, or in the general system.

These inter-reactions are exceedingly frequent. Indeed, it may be affirmed that no severe constitutional disorder can long continue in a woman during the predominance of the ovarian function without entailing disturbance in this function. And the converse is also true, that disorders of the sexual organs cannot long continue without entailing constitutional disorder, or injuriously affecting the condition of other organs.

These facts point to the necessity of guarding against the error of fixing our attention too specially upon one particular class of symptoms or upon one organ. Whilst searching out the part which is more especially the seat of diseased action, we must be careful not to overlook possible disease elsewhere, and not to neglect to observe the mutual reactions. The clinical physician, although led by the intuition of experience to seize quickly upon the offending part, will not omit to pass under review the state and working order of the other parts. In this manner most important complications are often brought most unexpectedly to light; and in every case some useful indication in treatment is discovered. The late Professor Chomel, a man of admirable skill, sagacity, and judgment, never failed, when a new case of disease came under his care, to interrogate successively every function. Thus, I have seen him in a case of pneumonia, the signs of which at once arrested attention, proceed nevertheless to explore the abdomen, the uterus, and the rectum. This may look like carrying out a principle to extremes. Yet who shall say that Chomel, as a clinical teacher or as a physician, was wrong?

It is not, indeed, necessary, in ordinary practice, to follow out in rigorous completeness the plan which to the clinical professor may seem desirable. It will therefore be useful to ascertain *what are the leading symptoms which, alone or grouped, indicate such disorder of the sexual organs as to call for direct exploration.*

This is the question we have set before us: When a woman presents herself, complaining of certain symptoms, chiefly subjective, some, or perhaps none, referred to the pelvis, how are we to act? Will these subjective symptoms enable us to refer them to their cause, to establish a diagnosis, to give satisfactory indications for treatment? Hardly. We must therefore call to our aid the objective signs; we must weigh and determine the significance of these before we can arrive at a conclusion at all precise or trustworthy as to the underlying pathological condition. The whole tendency of modern medicine is to subject every organ which manifests functional disorder to direct physical exploration, in order that it may solve the question presented obscurely by the subjective signs. The sound, the probe, the stethoscope, the laryngoscope, the otoscope, the ophthalmoscope, the various forms of speculum, are only so many contrivances for enabling us to project or extend the senses of touch, sight and hearing into the internal structures. In the case of the skin, all is at once exposed to direct observation; and, as Alibert remarked, we should be glad to have the same advantage in investigating and treating the diseases of the heart, lungs, liver, kidneys, and nervous centres. Why is it that the study of the pathology of the skin and of the eye is invested with such fascinating interest? Those who devote themselves with the greatest zeal and success to this study will tell you that it is because the skin and the eye reveal their condition directly to the senses, and thus furnish not only positive objective signs which the patient can neither suppress nor misrepresent, but also because in this direct observation of the skin and eye they can read and follow, as on a map or on a telegraphic dial, the working of distant organs and of many affections of the genera system. Here, then, you see how the reputed special practitioner, turning to account his special experience, often acquires an insight into general pathology denied to those who neglect the lessons they might read upon the visible organs.

This advantage we possess to a great extent of perfection in the case of the pelvic organs. It is by the proper use of this advantage that so great a degree of precision in knowledge, and of success in treatment of diseases of women, has of late years been attained. And there is one property in a high degree characteristic of the instruments employed in the investigation of the diseases of women of such singular value that it ought to completely silence the objections at one time so passionately urged against them. It is this: the instruments have a therapeutical as well as diagnostic application; the speculum, for instance, revealing a lesion of the cervix uteri, enables the surgeon at once to apply his remedy. Thus treatment follows upon the track of diagnosis, one sitting and one operation serving for both.

Here, then, as in medicine generally, our first indication of the direction in which we have to look for the disease which causes the patient to complain, lies in the disturbance of function. We have then to consider what these functions are. The first in importance, because it is continued with occasional interruptions throughout the period of active sexual life, is *menstruation.* The other functions are incidental to married life only; these are *the relation to the other sex*, *pregnancy*, and *lactation.*

Most of the diseases which attack the ovaries and uterus, whether primary or secondary, entail some disturbance in the menstrual function. The flow is diminished or in excess, or its periodicity is deranged. It is attended with pain in the pelvic organs, and other nervous phenomena.

We shall discuss the history of amenorrhœa, menorrhagia, and dysmenorrhœa hereafter. Our object now is simply to determine the conditions which suggest local examination. In the great majority of cases of *amenorrhœa* in single women, no local exploration is necessary; but in some cases it becomes imperative; for example, amenorrhœa is sometimes presumptive only—that is, the secretion takes place, but, owing to some imperfection of structure, it is retained in the cavity of the uterus or vagina. This may be called *occult menstruation.* The suffering becomes urgent in the highest degree, and nothing short of an operation which shall liberate the retained secretion will save the patient. Some cases, again, of suppressed menstruation, leading to effusion of blood behind the uterus, setting up circumscribed peritonitis, and displacing the uterus so as to press upon the bladder, may cause retention of urine. Here again, local examination is imperative. This may be said of every case of retention of urine. In almost every case of retention of urine in women the cause is external to the bladder, and in the great majority it is due to some disease or displacement of the uterus.

*Menorrhagia* is a relative term; that is, some women lose much more than the average without suffering in health; but whenever the loss continues profuse, obviously entails anæmia and general debility, and persists in spite of internal remedies, local examination is clearly necessary. We shall often find a sufficient local cause in polypus, tumor, inflammation, congestion, hypertrophy, displacement, or malignant diseases, all of which conditions require local treatment.

When we come to study the history of *dysmenorrhœa*, we shall find abundant proof of the almost constant association of this disorder with a mechanical condition of the uterus impeding the easy performance of the function. So long, however, as the distress does not clearly affect the general system, so long as it does not exceed endurable bounds, and if it appears to be moderated by general remedies, it is not necessary to examine; but, in the contrary event, examination should not be long postponed. To postpone examination is to postpone discovery of the cause and effective treatment. This is more especially imperative in the case of a married woman in whom dysmenorrhœa is complicated with dyspareunia and sterility. Abortion, if not primarily depending upon some local disease or displacement of the uterus, is so very likely to be followed by some such condition, that an examination should be instituted. If a sanguineous discharge, even periodical, resembling menstruation, goes on during lactation, especially if it be excessive in quantity and attended by leucorrhœal discharge, it may be almost confidently predicated that there exists some uterine disorder requiring local treatment.

I have used a new word, "*Dyspareunia.*" It is incumbent upon every one who coins a new word to explain its meaning and to justify the innovation. Just as the word "dysmenorrhœa" has been coined in order to express compendiously the condition of difficult or painful menstruation, just as "dyspepsia" is used to signify difficult or painful digestion—we want a word to express the condition of difficult or painful performance of the sexual function. Such a word would be convenient in many ways. It would enable us to avoid the longer and coarser forms in use, by substituting a single word, at once euphonious, expressive, and in harmony with medical language. After consulting with my colleague, Dr. W. H. Stone, whose high classical attainments give authority to his advice, I have determined to adopt the word "dyspareunia." It is derived from a Greek word, used in this sense by Sophocles. However disagreeable the topic may be, it is impossible to escape reference to a function so important. Dyspareunia in a female is perhaps the most absolute of all the indications of local malformation or disease. It calls the most imperatively for local examination as to its cause. In its milder forms it may make the sufferer's life a course of physical and mental wretchedness; in its severe forms it virtually unsexes her; and in any form it may lead to the most disastrous social calamities.

Taking this condition, dyspareunia, as a symptom of disordered function, we shall be astonished, when we proceed to direct examination of the organs concerned, at finding how many those causes may be, and what a wide field of pathological inquiry is associated with it. For example, there may be original defect or malformation; there may be obstructing tumors or growths, inflammation, dislocation or altered form, disordered innervation—in short, almost every disease to which the sexual organs are liable, may entail dyspareunia for one of its consequences; and in not a few of these diseases, disregard of this symptom may entail positive danger.

The existence of *certain discharges*, such as blood, under conditions of quantity and times of occurrence which distinguish it from normal menstruation, mucous, pus, albuminous, aqueous, fleshy, or membranous, if at all protracted, point clearly to some local disorder as their origin, which requires direct exploration.

Then there are some subjective signs, as pain, lumbo-dorsal, iliac, pelvic, or crural, and a sense of bearing down or pressure upon the rectum or bladder, entailing disorder in the function of these organs. These, especially if connected with abnormal discharges and other symptoms, call distinctly for local investigation.

Then we must observe the constitutional or remote effects of the foregoing conditions. Disorder of the pelvic organs seldom goes on long without entailing anæmia, disordered digestion, hyperæsthesia, neuralgia, or other manifestations of nervous derangement or prostration. Where these conditions are observed in association with marked signs of derangement of function of the pelvic organs, the necessity for exploring the physical state of these is as clear as is that of examining the state of the heart or lungs when these organs perform their function with distress, and the whole system suffers.

Such, then, is a summary view of the conditions, chiefly subjective, which point out to us the desirableness of instituting direct observation of the pelvic organs. This direct observation commonly enables us to analyze the groups of subjective symptoms; to determine the cause and significance of each, separately and collectively. It always brings to our assistance the discovery of other symptoms, entirely objective; and almost always puts it in our power to apply the proper treatment.—*Lancet*, Oct. 21, 1871.

### *Therapeutics of the Present Day.*

The *Practitioner*, for November last, contains some interesting observations on this subject, by Dr. Henry Kennedy, of Dublin. The leading points to which he calls attention are the following:

1. That the proper position the physician holds, in reference to the administration of drugs, is, that he treats, and, with the assistance of nature, cures disease by their means.

2. That in our endeavors to improve therapeutics, too much must not be expected, inasmuch as there is a limit beyond which we cannot pass, and this limit is, and must remain far short of certainty.

3. That if we ignore the labors of our predecessors we will commit a grave mistake; for they have left after them a mass of therapeutic facts which it would not be possible, even at the present day, to excel; and, therefore, our labors should begin where theirs ended.

4. That the physiological dose of each drug is the proper one to use, as it is only then its therapeutic virtues can be ascertained.

5. That at present the doses of many drugs are much smaller than our predecessors used; and, therefore, the results in our hands cannot be unsatisfactory.

6. That our predecessors, wherever it was possible, used medicines in the form of powder, which had the great advantage of being free from any risk likely to be caused by any other mode of preparation.

7. That experiments thus made must lead to more definite results than any made with other preparations of the same drugs.

8. That compound medicines, like the tincture of perchloride of iron, should be recognized as such, and not as simple drugs.

9. That the use of diluents is a very important principle to recognize in treating disease.

10. That a knowledge of human physiology is essential to give anything of a scientific status to our therapeutics.—*Virginia Clinical Record.*

***Transfusion.***

Dr. de Belina, of Heidelburg, ascribes, very justly, the failure of transfusion of blood, and the discredit into which it has fallen in France, mainly to the employment of blood which has not been defibrinated, to not determining the quantity of blood to be employed, and finally to the imperfection of the instruments and of the operative methods employed.

The use of blood, not defibrinated, inevitably results in its coagulation in the tubes of the instrument, hence the transfusion becomes impossible, or clots may be introduced into the vein, producing a dangerous or even fatal result. If the clots be large, obstruction of the pulmonary artery and immediate death may result; and if death be not immediate, it may result from an embolus produced by the deposit of clots in some portion of the circulatory apparatus.

Fibrin is not an essential part of the blood, and it may be removed without inconvenience; and, further, the process by which the blood is defibrinated improves it by saturating it with oxygen, and by removing the carbonic acid.

As to the quantity, either too much is often employed, or too much at a time, whence results afflux to the heart, consecutive paralysis, or at least dangerous congestions in different organs.

A proper apparatus for transfusion should fulfill the following conditions:

1st. It should be capable of being kept perfectly clean.

2d. It should have sufficient capacity to contain the required amount of blood, and it should be easily used.

3d. The blood in it should be maintained of the desired temperature.

4th. The introduction of bubbles of air into the vein should be impossible.—*Revue de Therap.*, Oct. 15, 1871, from *Receuil d Med Vet.*—*Virginia Clinical Record.*

***Bromide of Calcium as a Nervine.***

According to Dr. William A. Hammond (*Boston Med. and Surg. Jour.*), "Bromide of calcium is a white crystalline substance, very soluble in water, and readily decomposing on exposure to the atmosphere for a few minutes. The aqueous solution is at first colorless, but it soon becomes tawny from a portion of the bromine being set free. Its taste is similar to that of the bromide of potassium, though somewhat more pungent and disagreeable. The formula of bromide of calcium is Br. Ca., and its combining equivalent is 98 (Br. 78, Ca. 20=98); 100 grains, therefore, contain about 79.5 grains of bromine. The dose is from fifteen to thirty grains or more for an adult. It is especially useful in those cases in which speedy action is desirable, as, owing to its instability, the bromine is readily set free, and its peculiar action on the organism obtained more promptly than when either of the other bromides is administered. Chief among these effects is its hypnotic influence; and hence the bromide of calcium is particularly beneficial in cases of delirium tremens, or in the insomnia resulting from intense mental labor or excitement. Thus, I gave a gentleman who, owing to business anxieties, had not slept for several nights, and who was in a state of great excitement, a single dose of thirty grains. He soon fell into a sound sleep, which lasted for seven hours. The next night, as he was wakeful, I gave him a like dose of bromide of potassium, but it was without effect, and he remained awake the whole night. The subsequent night he was indisposed to sleep as he had ever been, but a dose of thirty grains of bromide of calcium gave him eight hours of sound sleep, and he awoke refreshed, and with all unpleasant cerebral symptoms—pain, vertigo, and confusion of ideas—entirely gone. In a number of other instances, a single dose has sufficed to induce sleep, a result which rarely follows the administration of one dose of any of the other bromides. In some exhausted conditions of the nervous system, attended with great irritability, such as are frequently met with in hysterical women, and which are indicated by headache, vertigo, insomnia, and a mental condition of extreme excitement, bromide of calcium has proved in my hands of decided service. Combined with the syrup of the lacto-phosphate of lime, it scarcely leaves anything to be desired. An eligible formula is—R. Calcii bromidi 1 oz.; syrup. lact. phos. cal. 4 oz. M. ft. sol. Dose, a teaspoonful three times a day in a little water. In epilepsy I have thus far seen no reason for preferring it to the bromide of potassium or sodium, except in those cases in which the paroxysms are very frequent, or in cases occurring in very young infants; of these latter, several, which had previously resisted the bromide of potassium, have yielded to the bromide of calcium. It does not appear to cause acne to anything like the extent of the bromide of potassium or of sodium."

***Symmetry in Cutaneous Affections.*** — Clinique of Mons. Noel Gueneau de Mussy.

The tendency to symmetry, which is one of the laws of normal organic development, is often found as a pathological symptom, and manifests itself by the repetition of the same morbid action in similar portions of the two sides of the body. Thus the caries of one tooth is often followed by the caries of the corresponding tooth; the affections of the eyes are often double; the wound of one eye, especially when a foreign body has penetrated the organ, frequently involves the other. In gout and in rheumatism, very often, when one articulation is attacked, the corresponding one is involved at the same time or is invaded soon afterwards.

In eruptive fevers, in many cutaneous affections, this symmetry is observed; it is more easily explained when occurring in organs to which are distributed the cerebro-spinal nerves, which are developed symmetrically on the two sides of the body, and which are under the control of the strongly centralized innervation whose source is the encephalon. It is there more apparent; it shows itself, however, in the domain of the grand sympathetic. Here also are pathological consensus, sympathies in the proper sense of the word between the two halves of simple organs, between the double organs, like the two kidneys, the two ovaries.

Graves has described one of the most striking examples of pathological symmetry in median erysipelas. He has formulated this law, that when erysipelas commences on the median line, it will develop itself symmetrically on each side. A case observed recently by myself at the Hotel Dieu, gives such a striking and curious confirmation of this law of Graves' that I think it proper to report it.

During June, 1871, I received into my wards a man affected with erysipelas of the face. The attack had commenced on the bridge of the nose, and had extended symmetrically on both sides. The third day it occupied the brow, but beyond and beneath the frontal protuberances there existed two triangular spaces nearly two and one-half centimetres, where the skin, pale and depressed, remained perfectly sound. Its color, normal in this space, contrasted with the red carmine coloration of the neighboring parts. The edges which surrounded this part of the skin, still unattacked by the erysipelas, formed a raised border, indicating, according to the remark of Chomel, that the morbid action had not been arrested, and that this portion of the teguments, as yet respected, would undergo an ulterior invasion.

On the left the external border of the triangle corresponded exactly to a linear cicatrix caused by an ancient wound of the brow which had divided the skin in its entire thickness. The right half of the brow had not received any injury, but nevertheless the part of the integuments untouched by the erysipelas presented exactly the same form, the same seat and the same dimensions as that which in the left side was contiguous to the cicatrix. These two triangles had a perfect geometrical equality; their position and direction were absolutely similar.

We may suppose that the interruption of the vessels by the cicatrix, which was evidently perpendicular to the branches of the frontal artery, had retarded on the left the erysipelatous action, but on the right the law of symmetry alone was able to explain this anomaly which was only temporary as I had forseen. At the end of twenty-four hours the erysipelas had passed the obstacle and the brow presented a uniform redness, upon which the cicatrix of the left side marked only by a white line the place which the previous evening had exhibited the triangle.

The seventh day the erysipelas had disappeared and the patient became convalescent.

This case, confirming the law of Graves, appeared to me to raise interesting questions of pathological physiology. What is the cause of this unison which clothes the morbid action with such vigorous equality and such perfect symmetry between similar parts? If, as physiology teaches, the vascular action which controls the extension of the erysipelas is placed under the operation of the nervous system, we may say that the left centripetal nerves have transmitted to the centres of innervation the impression of the obstacle presented by the cicatrix to the advance of the erysipelas, and that these centres had controlled, in consequence of the vaso motor, innervation necessary to harmonize the two sides.

It is certain that the sympathetic nerves anatamose among themselves, that the two halves of the brain are united by numerous commissures; perhaps, even as some anatomists admit, the cerebral nerves right and left are reunited near their origin. But in what conditions and in what manner is the action of these nerves harmonized? Our observations and our scalpels have as yet interrogated the cerebral mass without discovering the secret.

In some cases we are able to establish a connection between the anomalous modifications of the nerves and the lesions of the integuments. Thus we sometimes observe violent pains in the lumbar region towards the emergence of the nerves, where some days afterwards the appearance of zona (shingles) follows the direction, and indicates, so to speak, the route. Often it even throws its vesicular groups over the track of the different branches which have among themselves connection in their origin. An eruption may succeed the neuralgias, which are sometimes very obstinate. I remember having seen, with Chomel, an old lady who, twelve or fifteen years previously, had been affected with zona, and who suffered, many times a year, attacks of violent neuralgia, occupying the region where the hepatic groups had been developed.

I have elsewhere cited the singular excitability of the skin by certain agents in patients attacked by neuralgia. In two of these patients of whom I have spoken, opium, and in others turpentine, produced the appearance of eczematous eruptions upon the parts whose sensibility had been altered.—*Revue Medicale.—Virginia Clinical Record.*

### *Carbolic Acid and Sulphite of Soda in Small Pox.*

Dr. D. P. Boyer, of Phila., states (*Med. and Surg. Reporter*), that the following treatment has proved as much a specific in small pox as quinia is in intermittent fever; not only in his hands but also in those of Dr. A. H. Boyer, of Bridesburg.

"Considering *small pox* purely a *blood poison*, and the eruption an effort of nature to throw off or eliminate that poison, I concluded to strike at the root of the disease, and direct my treatment solely to the eradication of that poison. For which purpose, I gave a solution of 2 grains of carbolic acid, and 15 or 20 grains of sulphite of soda every three (3) hours, *with no other* treatment than an ordinary purge during the initiative or forming fever. The result, after several months' trial with myself and son, has been that in *every case of variola* and confluent small pox, on the fourth day of the eruption, the swelling of the face abated, the pulse fell to a normal rate, and the tongue commenced cleaning, the eruption commenced to dry up, and the pustules withered and *shriveled.* By the seventh and eighth day of the eruption the patient was convalescent, without a sign or mark of having small pox after the slight desquamation of the light scales, or scales fell off. In no case by this treatment did the pustules positively mature, but always dried up before maturation. Externally, any soothing or cooling application for the first three days is all that is required to allay the itching, etc.

"N. B.—I am not satisfied that this treatment is a protection from future attacks of small pox, from the fact that it eliminates the poison from the blood, and dries up the eruption, etc. I believe that it leaves the system in exactly the same condition in which the disease found it. I think vaccination is required after the disease."

### *Excision of the Shoulder-Joints for Diseases.*

Dr. Ewens, of Cernes Abbas, reports in the *Lancet* two cases of diseases of the shoulder, in one of which an elliptical incision was made, whilst in the other excision was performed by a modification of the single longitudinal incision, both terminating successfully. The first case occurred in a girl, aged 18, who originally suffered from inflammation, apparently of a rheumatic character, of the left shoulder-joint. An abscess formed and pointed at the posterior border of the insertion of the deltoid muscle. Six months after, she was weak and emaciated, with almost complete loss of power to move the arm herself, and forced movement excited great pain. There was a second opening above the joint, when the abscess burst, and both communicated with a sinus leading to the posterior portion of the axilla. No diseased bone could be detected by a probe, but it was shrewdly suspected that disease of the shoulder-joint existed. She was placed on a generous diet, cod-liver oil and iron. The paroxysms of pain were very severe, and as the disease advanced, became so violent as to necessitate a frequent resort to hypodermic injections.

At length, acting on the advice of Mr. Pollock, Dr. Ewens made an exploratory incision, and after some groping discovered loose bone. The ordinary elliptical incision was thus made, the flap dissected up, and the diseased joint fully exposed. The head of the bone was found in a very carious condition, and a portion, shortening the bone by about an inch, was removed by Butcher's saw. The wound was sponged with carbolic acid and oil, in the proportion of one part of the acid to four of the oil; the flaps re-adjusted and secured by pins and twisted suture. Sickness, due to the chloroform, occurred after the operation, but passed off in the course of 24 hours. She progressed favorably for 10 days, when a slight rheumatic attack supervened, which was cured by ammonio-citrate of iron and bicarbonate of potash. From this date she gradually improved; but a small sinus still remained, which, indicating more diseased bone, necessitated a further operation. A small portion of the shaft was again removed, and rapid recovery followed.

Fourteen months afterward the arm was two inches shorter than the other. There was perfect use of the arm for underhand work, and power to move it behind her and to bring it forward on the chest. There was, however, no power to raise the arm.

In the second case, a large abscess had formed beneath the right pectoral, and had been opened; a sinus remained beneath the outer part of the mammæ, behind the pectoral muscle, into the axilla, but a long probe failed to reach diseased bone, or to find the end of the sinus. Careful examination elicited the history of a blow over the front of the shoulder three years prior to the opening of the abscess, with an account of symptoms of joint disease, but ascribed to rheumatism, in the intermediate period. A swelling was found on the back of the shoulder, and opened by Dr. Ewens, and curdy pus was evacuated. Her general health improved, but several times spots of erratic erysipelas appeared on the arm, speedily subsiding under the local application of strong tincture of iodine. As the sinuses did not heal up, an exploratory incision was determined upon, and the wound at the back of the joint was enlarged so as to enable the finger to be introduced under the deltoid, which was then cut through transversely, a little below its origin from the spine and acromion process of the scapula, and the joint was thus laid open posteriorly.

There was little hemorrhage. The head of the bone was found to be completely carious, with a large sequestrum in its centre. The posterior half of the deltoid being thus divided horizontally, a perpendicular incision carried through its whole length down to its insertion into the humerus, fully revealed the parts to be removed. The patient being very fat, Butcher's saw could not be conveniently used, and the bone was therefore sawn through with an ordinary finger saw, the portion removed representing a shortening of about two inches. The wound was dressed as in the former case, and the patient made a more than ordinarily quick recovery, she being perfectly well in three months.

***Disinfectants.***

M. Gille has published in the *Archives Medicales Belges*, an interesting article "On the value of a Disinfectant," in which he says we must not only get rid of offensive smells, but of all other products of decomposition, and that any substance which only effects one of these ends, is a very imperfect disinfectant. He then passes in review some of the disinfectants in common use. Sulphate of iron he considers is useful from its action in decomposing ammonia, carbonate, and sulphohydrate. Perchloride of iron, besides this, precipitates albuminoid matters, and acts also by its chlorine. Lime disinfects organic matters, fixing carbonic acid and sulphuretted hydrogen, and decomposing hydrosulphate of ammonia. The permanganate of potass. is a most energetic oxidizing agent, decomposing sulphuretted hydrogen, destroying organic matter, and acting upon all fixed compounds with which it comes in contact.

It may be remembered that M. Decaisne employed it in dissecting rooms, but that M. Gosselin, in 1864, reported that it was not adapted for this purpose. Chlorate of potassi may be used to disengage chlorine in places that are not easy to reach by other means. This is a capital plan for cesspools and middens.

Chloride of lime acts by the chlorine it sets free, and chemically decomposes most foul gases. M. Decaisne considers it the best disinfectant of offensive gases. Does it also, mixed with metallic oxides, act by disengaging oxygen as has been asserted? M. Gille doubts this. He also observes that, although chloride of lime destroys offensive gases, it does not arrest putrefaction, but by the lime set free, hastens the process.

Hydrochloric acid is employed to disinfect dog-kennels. Vessels containing it being left open, it completely destroys the offensive gases that abound where a large number of dogs are kept. This plan is adopted in the Veterinary School of Brussels.

The action of carbolic acid, M. Gille says, is not chemical. He accepts what is commonly called the germ theory, inasmuch as he says the acid prevents germs from provoking putrefaction. He also thinks it will hinder the formation of miasms, and is, therefore, a good preventive of epidemics. It is thus to be seen that all the disinfectants are good, but that they should be used with discernment, a selection being made according to the products we wish to get rid of.—*Canada Med. Journal.*

